# Clinical effectiveness of nimodipine for the prevention of poor outcome after aneurysmal subarachnoid hemorrhage: A systematic review and meta-analysis

**DOI:** 10.3389/fneur.2022.982498

**Published:** 2022-09-21

**Authors:** Guangzhi Hao, Guangxin Chu, Pengyu Pan, Yuwei Han, Yunzheng Ai, Zuolin Shi, Guobiao Liang

**Affiliations:** Department of Neurosurgery, General Hospital of Northern Theater Command, Shenyang, China

**Keywords:** subarachnoid hemorrhage, nimodipine, meta-analysis, poor outcome, mortality, cerebral vasospasm

## Abstract

**Objective:**

In clinical practice, nimodipine is used to control cerebral vasospasm (CVS), which is one of the major causes of severe disability and mortality in patients with aneurysmal subarachnoid hemorrhage (aSAH). However, the exact efficacy of nimodipine use for patients with aSAH is still controversial due to the lack of sufficient and up-to-date evidence.

**Methods:**

In this meta-analysis, the latest databases of the Cochrane Central Register of Controlled Trials, PubMed-Medline, Web of Science, Embase, Scopus, and OVID-Medline were comprehensively searched for retrieving all randomized controlled trials (RCTs) regarding the efficacy of nimodipine in patients with aSAH. The primary outcome was a poor outcome, and the secondary outcomes were mortality and cerebral vasospasm (CVS). After detailed statistical analysis of different outcome variables, further evidence quality evaluation and recommendation grade assessment were carried out.

**Results:**

Approximately 13 RCTs met the inclusion criteria, and a total of 1,727 patients were included. Meta-analysis showed that a poor outcome was significantly reduced in the nimodipine group [RR, 0.69 (0.60–0.78); I^2^ = 29%]. Moreover, nimodipine also dramatically decreased the mortality [RR, 0.50 (0.32–0.78); I^2^ = 62%] and the incidence of CVS [RR, 0.68 (0.46–0.99); I^2^ = 57%]. Remarkably, we found a poor outcome and mortality were both significantly lower among patients with aSAH, with the mean age < 50 than that mean age ≥ 50 by subgroup analysis. Furthermore, the evidence grading of a poor outcome and its age subgroup in this study was assessed as high.

**Conclusion:**

Nimodipine can significantly reduce the incidence of a poor outcome, mortality, and CVS in patients with aSAH. Moreover, we strongly recommend that patients with aSAH, especially those younger than 50 years old, should use nimodipine as early as possible in order to achieve a better clinical outcome, whether oral medication or endovascular direct medication.

**Systematic review registration:**

www.york.ac.uk/inst/crd, identifier: CRD42022334619.

## Introduction

Cerebral vasospasm is still the major cause of severe disability and mortality in patients with aneurysmal subarachnoid hemorrhage (aSAH), which accounts for 3–5% of all strokes and annually poses a life and health threat to 600,000 persons worldwide (1). It was worth noting that the combined morbility and mortality in younger patients with aSAH reaches as high as 50% (1, 2). Moreover, Lashkarivand et al. have reported that the 1-year mortality rate of patients with aSAH can reach 65.8%, even with the early aneurysm clipping or interventional embolization (3–5). Therefore, it is urgent for a clinical expert to identify an effective drug to prevent patients with aSAH from CVS and subsequent serious adverse outcomes, including severe disability and mortality.

Some studies have previously reported that nimodipine had a moderate effect of reducing cerebral vasospasm in patients with aSAH (6, 7). However, due to the limited research literature and insufficient updated evidence, there is still not a unified and precise conclusion on the efficacy of nimodipine in SAH. For example, whether the route of nimodipine administration has an impact on the final outcome, whether the patients of different ages have variable responses to nimodipine, and whether the results of multi-center studies and single-center studies are consistent have not been comprehensively investigated and elaborated in previous pieces of literature.

In the last decade, four updated RCTs investigating the effect of nimodipine on the clinical outcome have become available (8–11). In these trials, the route of drug administration and dose and control measures was different from previous studies. Therefore, it is essential to conduct a comprehensive meta-analysis, including all RCTs, for better revelation of the medical efficacy of nimodipine in treating patients with aneurysmal SAH. Currently, there is no definitive evidence of recommendation for the clinical application of nimodipine in patients with aSAH. Given the circumstances, our detailed and comprehensive meta-analysis study of nimodipine might provide a valuable guidance for its accurate use in the clinical treatment of patients with aSAH.

## Methods

### Protocol and guidance

**Figure 1:**
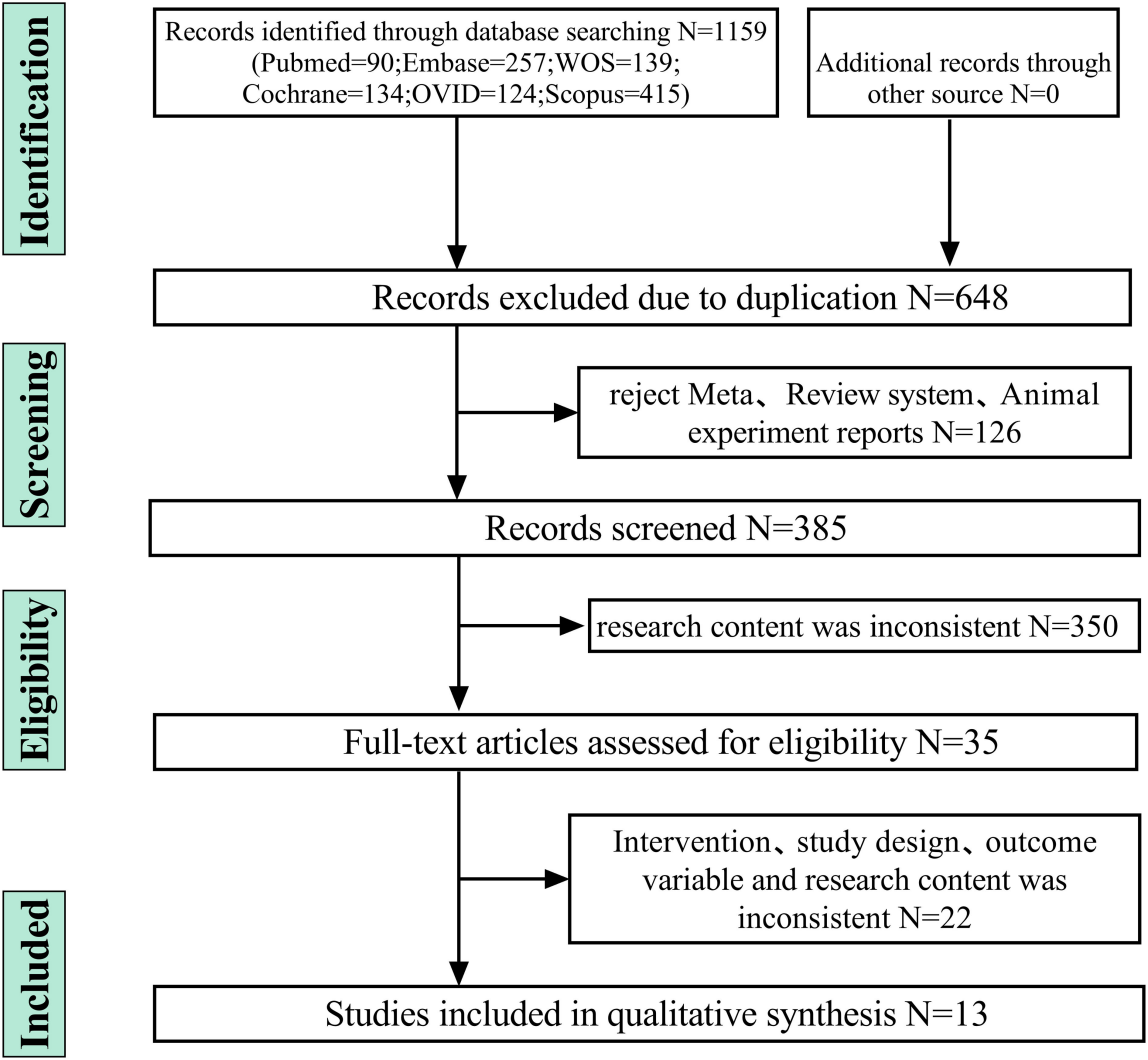
Identification and screenning strategy of final included and excluded studies.

This study was performed in accordance with Preferred Reporting Items for Systematic Reviews and Meta-Analysis (PRISMA) (12). The flowchart of the literature search strategy is presented in Figure 1. In the initial search, the discrepancies were resolved by two researchers (CGX and SZL). The search strategy is presented in Supplementary Table 1. The protocol for this review was registered with PROSPERO (CRD42022334619).

### Inclusion criteria

Trials were considered to be eligible if they met the following criteria: (1) randomized controlled trials; (2) age ≥ 18 and no sexual or racial restrictions; (3) aSAH diagnosed using computed tomography or lumbar punctures; (4) patients treated with nimodipine; and (5) outcomes data, including severe disability, mortality, and cerebral vasospasm.

### Exclusion criteria

Reports were excluded if they had met the following criteria: (1) animal experiments; (2) SAH due to other etiologies, not intracranial aneurysm; (3) no unambiguous definition and score provided for severe disability, mortality, and cerebral vasospasm; (4) Studies were excluded if low-dose nimodipine was compared with high-dose nimodipine; and (5) clinical observations, reviews, and trials without detailed statistical analysis.

### Outcomes

The primary outcome was a poor outcome. Secondary outcomes were mortality and cerebral vasospasm. Supplementary Table 2 shows the definitions of these outcome variables.

### Search strategy

Several chief databases were retrieved by one of the authors (HYW), including Pubmed-Medline, Embase, Web of Science, Scopus, Ovid-Medline, and the Cochrane Central Register of Controlled Trials (CENTRAL). The ongoing or unpublished eligible trials were identified by searching the ClinicalTrials.gov. Supplementary Table 1 presents the search strategy.

### Study selection

After removing duplicates, animal studies, and reviews, two independent researchers (CGX and SZL) screened all titles and abstracts. Finally, complete pieces of literature were obtained for further screening. Inconsistencies were resolved through collective discussions.

### Data collection process

The data from the final included trials were extracted by two independent researchers (AYZ and HGZ) by using a standard data extraction form, including authors, publication time, population characteristics, study design, interventions, sample size, outcome indicators, and follow-up time. The primary outcomes were poor outcomes defined as a modified Rankin scale (mRS) score of 4–6 or a Glasgow Outcome Score (GOS) of 1–3 (13, 14). The secondary outcomes were mortality and cerebral vasospasm.

### Assessment of risk of bias

We used the Cochrane Collaboration risk of bias tool to assess the quality of all included trials by two researchers (HGZ and AYZ) independently (15).

### Quality of evidence and recommended strength

The grading of recommendations assessment, development, and evaluation (GRADE) approach was used to examine the quality of evidence, including the three outcomes and their subgroup analysis results (15, 16).

### Data synthesis and sensitivity analyses

We performed literature statistical analyses using Stata 16.0 (StataCorp LP) and RevMan (version 5.3; The Cochrane Collaboration). Risk ratios and their associated 95% confidence intervals were used to assess outcomes, and the *P*-value < 0.05 was considered to be statistically significant. We used the inconsistent index (I^2^) test to assess heterogeneity among the studies, and both *p* > 0.1 and I^2^ < 50% were considered to indicate no heterogeneity (17). The fixed effects models were used to pool outcomes if significant heterogeneity was not present (I^2^ < 50%). Otherwise, we used the random effects models when significant heterogeneity was present (I^2^ ≥ 50%). The sensitivity analysis was used to determine the stability. The possibility of publication bias of included trials was shown by the funnel plot and further quantitatively assessed by Egger's test and Begg's test (18).

### Subgroup analyses

Subgroup analyses were performed to test interactions between two subgroups according to the mean of age (≥50 and <50), administrations (oral and vessel), sample size (≥80 and <80), and the number of the research centers (single center and multi-center). Detailed subgroup analyses were performed for the two variables of a poor outcome and mortality.

## Results

### Eligible studies and study characteristics

We initially identified 1,159 records and included 13 eligible trials in the final meta-analysis (Figure 1) (8–11, 19–27). Supplementary Table 3 shows study and population characteristics of included trials. These trials comprised 1,727 participants, with 561 severe disability and 293 deaths.

**Figure 2:**
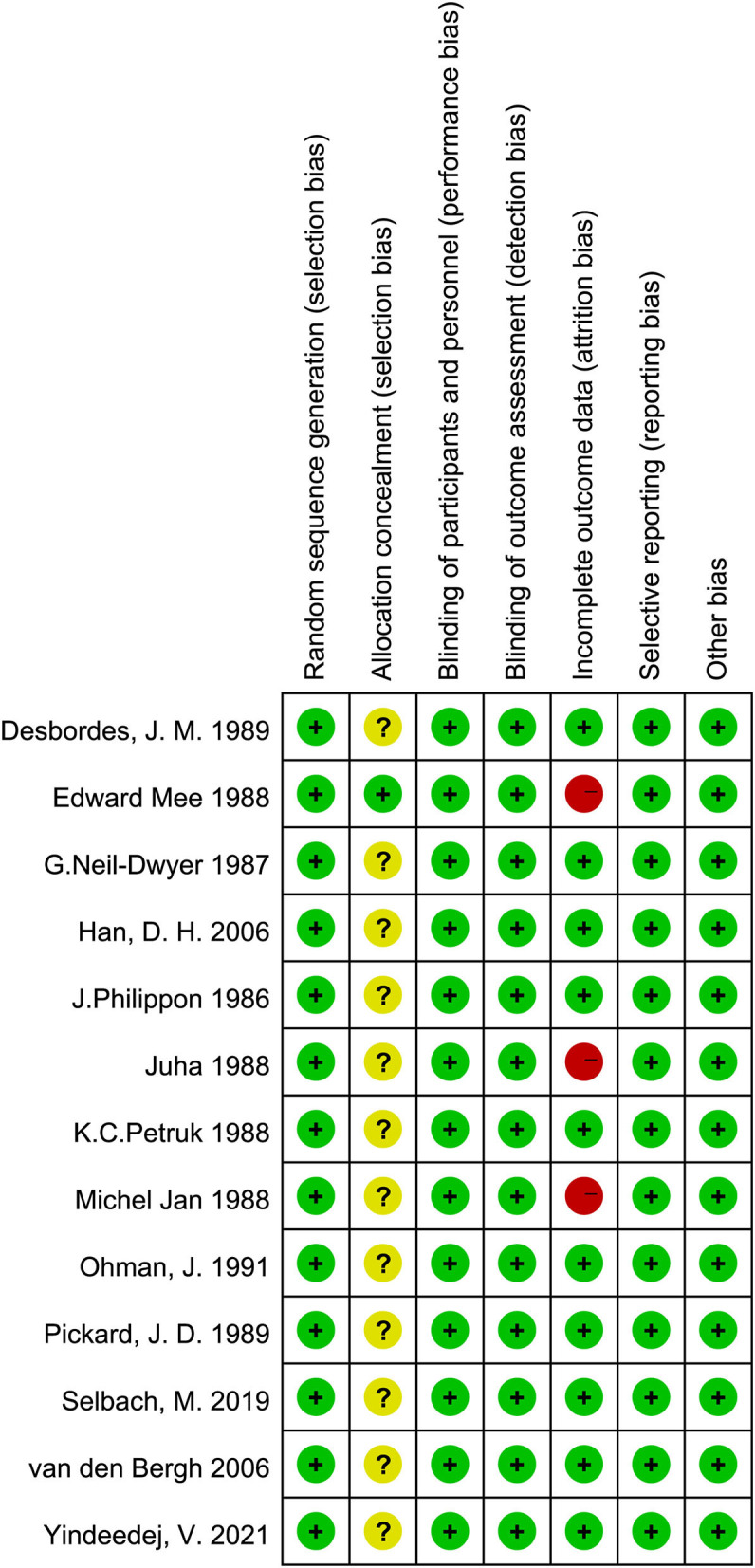
Quality of the included RCTs assessed by Cochrane Risk of bias summary.

**Figure 3:**
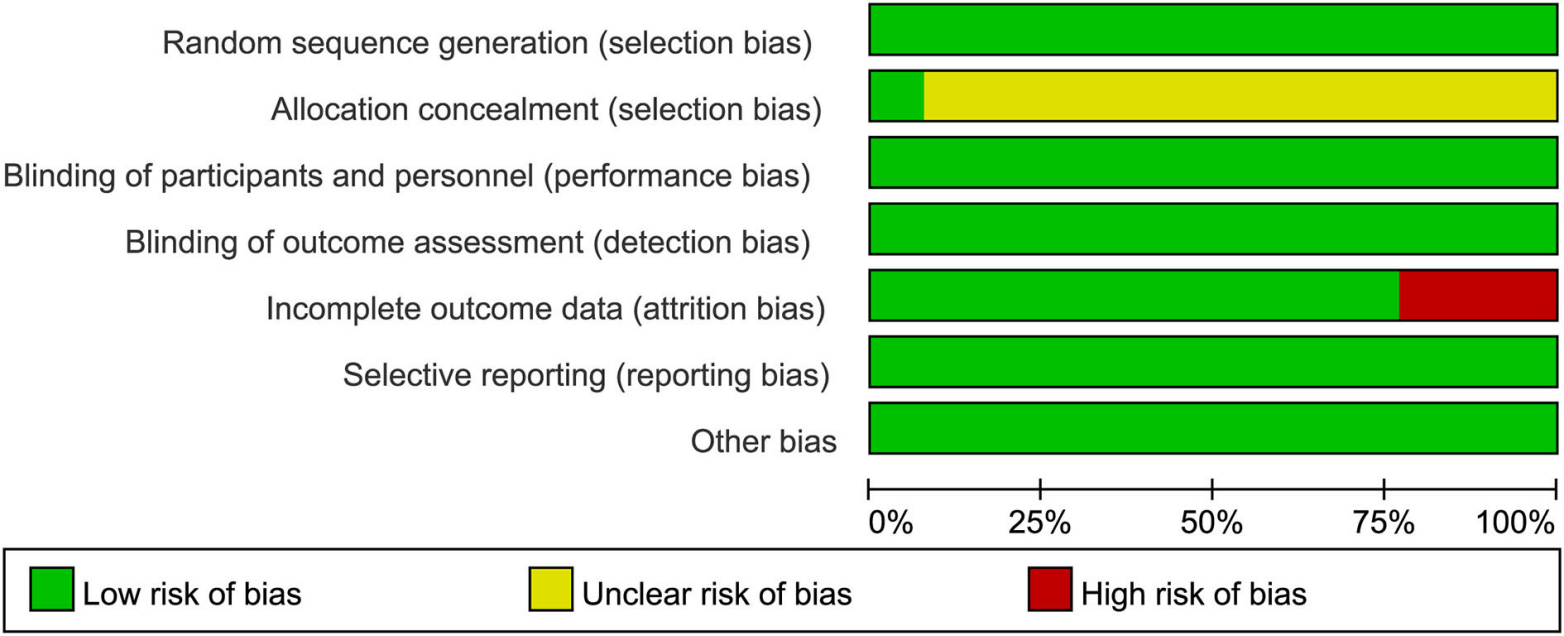
Quality of the included RCTs assessed by Cochrane Risk of bias graph.

Figures 2, 3 show the risk of bias. Using the GRADE summary of evidence, the quality of evidence and recommended grade for each outcome variables and their subgroup results are detailed in Supplementary Table 4 (15, 16).

### Primary outcome: Poor outcome

**Figure 4:**
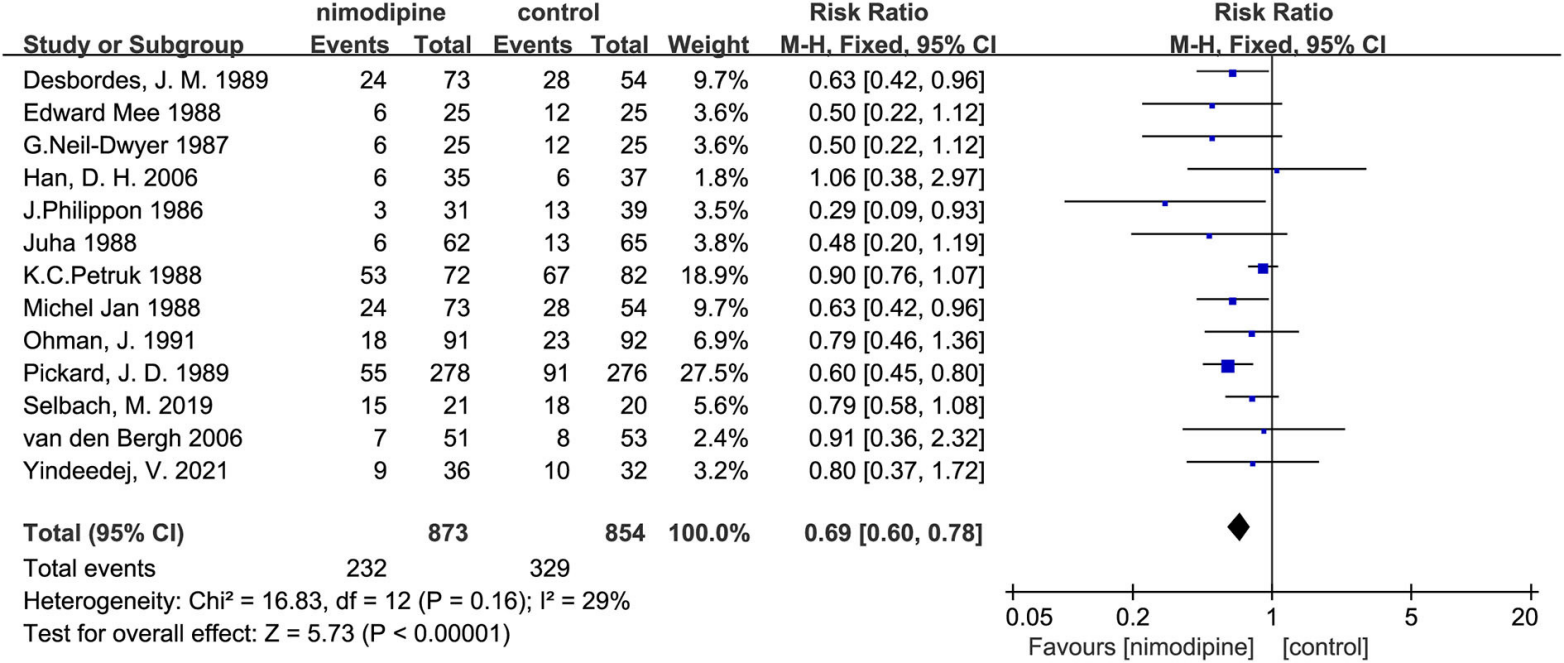
Efficacy of nimodipine-use in the prevention of poor outcome in SAH patients.

**Figure 5:**
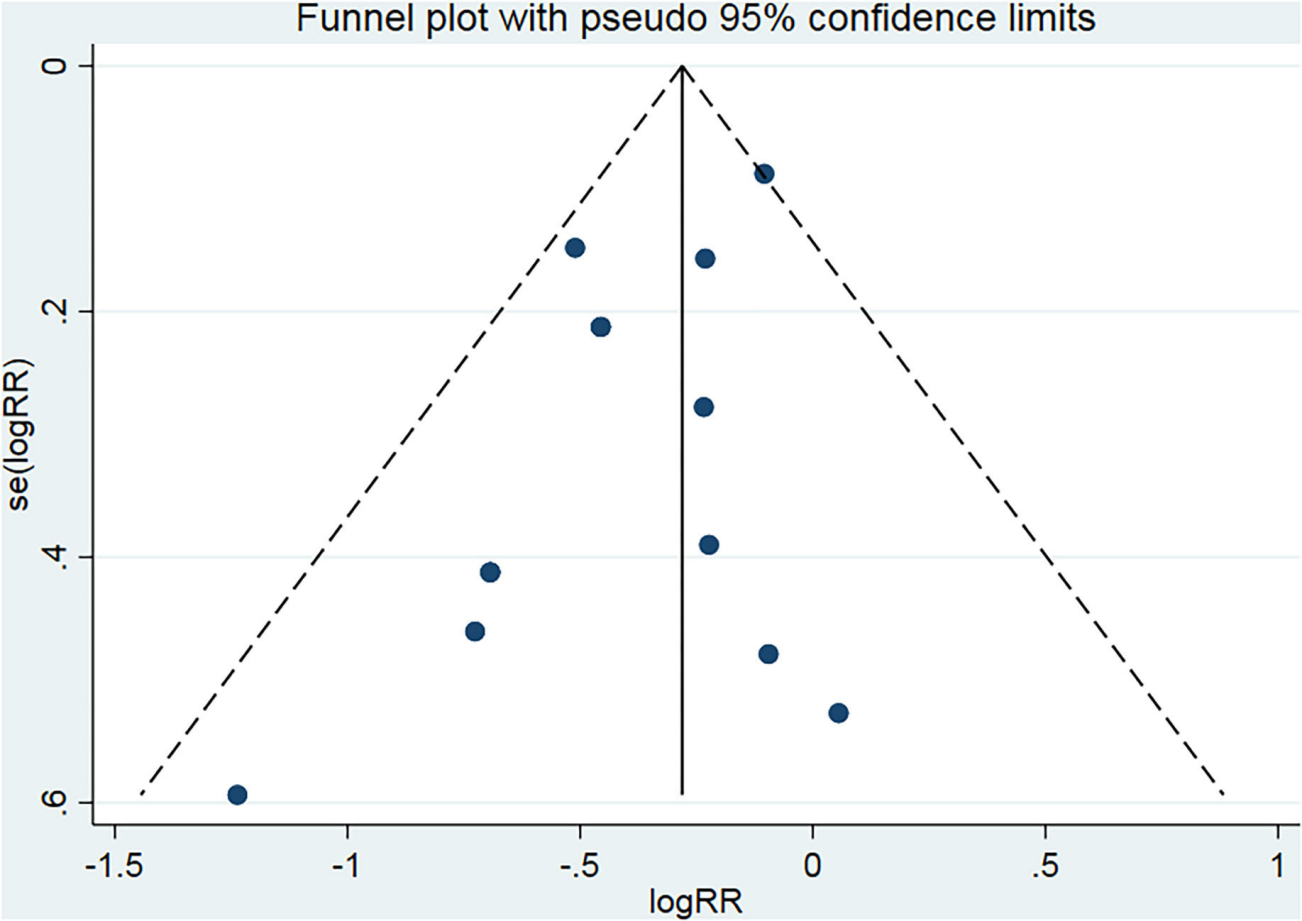
Funnel plot for the bias assessment. RR, relative risk; SE, standard error.

All 13 trials reported severe disability and the mortality. The poor outcome was calculated as the total number of severe disability and the mortality. There was significantly statistical difference in the poor outcome between the nimodipine group and the control group (RR = 0.69, 95% confidence interval, 0.60–0.78, I^2^ = 29%; Figure 4). Funnel plot analysis showed no asymmetry (Figure 5). Additionally, Egger's test (*p* = 0.051) and Begg's test (*p* = 0.669) showed no publication bias.

After deleting any one of the documents, the combined effect sizes of all the remaining pieces of literature were between 0.64 and 0.72. There was no apparent difference among these results, which revealed that all the literature included in this meta-analysis passed the sensitivity test.

**Figure 6:**
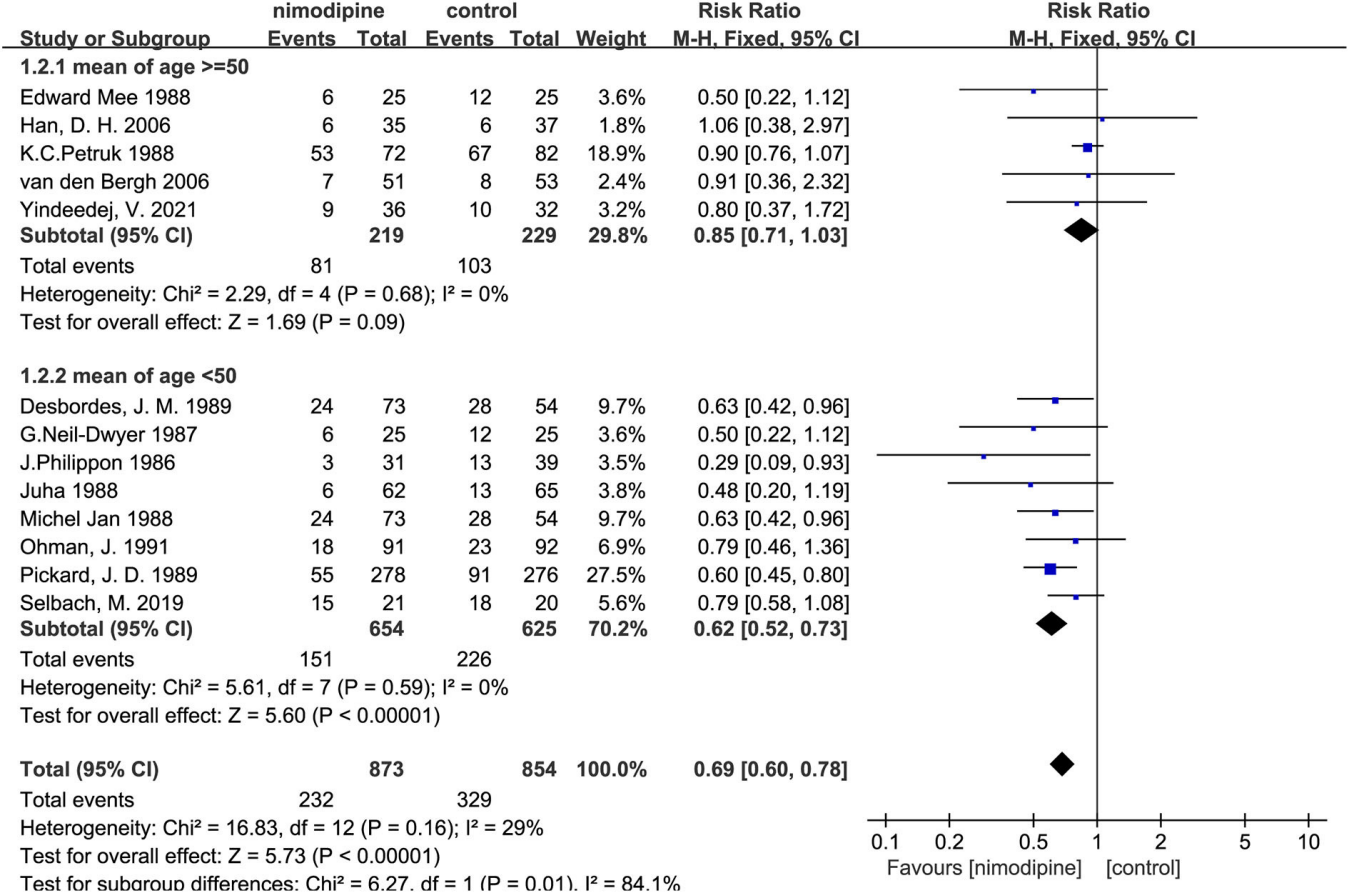
Subgroup analysis of poor outcome according to the mean age.

Subgroup analysis found that the poor outcome was significantly lower among the patients with the mean age < 50 than that mean age ≥ 50 (*P* for interaction = 0.01; Supplementary Table 5; Figure 6). Nevertheless, no significantly statistical difference was found in other three subgroups of sample size, the number of research centers, and the route of drug administration (Supplementary Table 5).

### Secondary outcome: Mortality and cerebral vasospasm

**Figure 7:**
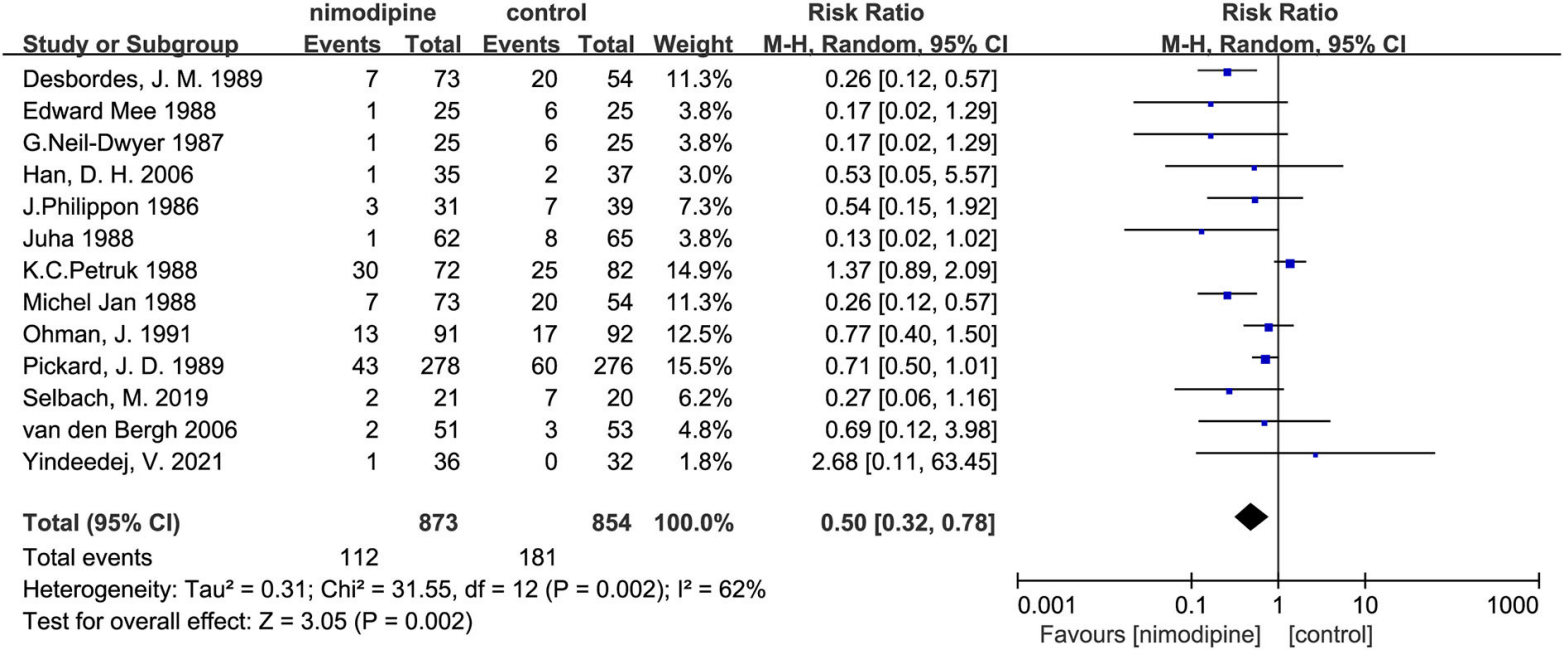
Efficacy of nimodipine-use in the prevention of mortality in SAH patients.

**Figure 8:**
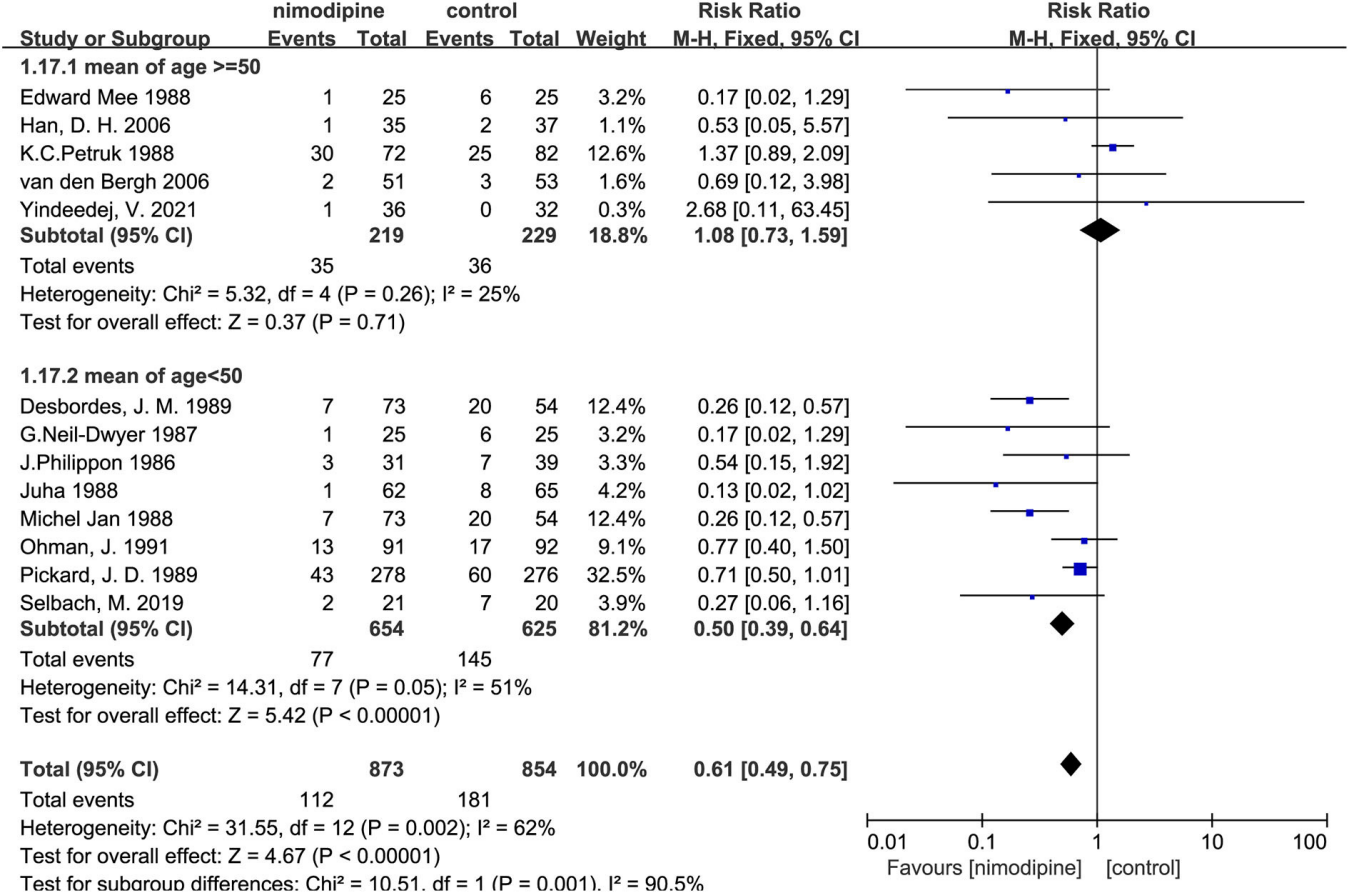
Subgroup analysis of mortality according to the mean age.

Nimodipine could significantly reduce the mortality of patients with aSAH than that in control group (RR = 0.50, 95% confidence interval, 0.32–0.78, I^2^ = 62%, *p* = 0.002; Figure 7). Further subgroup analyses showed that the mortality was significantly lower among patients with the mean age < 50 than that mean age ≥ 50 (P for interaction = 0.001; Figure 8; Supplementary Table 6). Similar to the results of the three subgroup analyses with a poor outcome, there was no significantly statistical difference among the sample size, the number of research centers, and the route of drug administration subgroups (Supplementary Table 6).

**Figure 9:**
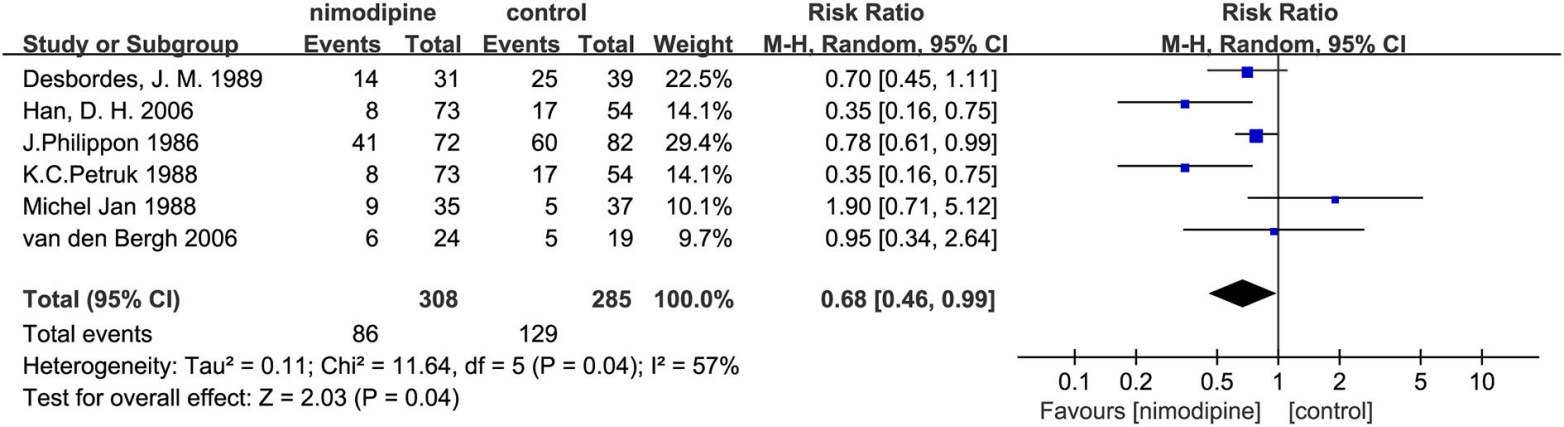
Efficacy of nimodipine-use in the prevention of cerebral vasospasm in SAH patients.

Furthermore, we found statistically significant difference in the incidence of cerebral vasospasm between the nimodipine group and the control group (RR = 0.68, 0.46–0.99, I^2^ = 57%, *p* = 0.04; Figure 9).

## Discussion

In this meta-analysis of 13 randomized controlled trials, with a total of 1,727 participants, the use of nimodipine was significantly associated with a poor outcome (RR = 0.69, 95% confidence interval, 0.60–0.78, *p* < 0.05) and mortality (RR = 0.50, 95% confidence interval, 0.32–0.78, *p* = 0.002 < 0.05). Moreover, the findings also suggested that nimodipine could significantly reduce the incidence of cerebral vasospasm in patients with aSAH after the meta-analysis of 6 RCTS (RR = 0.68, 95% confidence interval, 0.46–0.99, *p* = 0.04 < 0.05).

### Principal findings and comparison with other studies

The methods of this study on a poor outcome differed from one previous meta-analysis. A system review in 2011 found that nimodipine could decrease patient death by 74% in analyses of 8 trials with a total of 1,514 participants (*p* = 0.008, OR = 0.26, 95% CI, 0.09–0.71) (28). The previous review has probably reached more broad conclusions as a result of inaccurate prognostic evaluation methods and insufficient updated published trials. We expanded the scope of the database and found three earlier pieces of literature that met our inclusion criteria (21–23). Compared with the previous review, we excluded three trials without detailed GOS or WFNS scoring system and included four recent updated RCTs published after 2005, involving different control measures and routes of administration, so that the proportion of the latest pieces of literature has increased by 31% (8–11).

Moreover, previous research mainly focused on the analysis of the overall mortality in patients with aSAH (28). However, in addition to this main variable of a poor outcome, our study went further to provide a comprehensive analysis of the incidence of the mortality and cerebral vasospasm. Statistical results combined with severe disability and mortality could more accurately reveal the efficacy of nimodipine in patients with aSAH. Therefore, we chose the poor outcome as the primary outcome calculated by the sum of severe disability and death.

In this meta-analysis, we found that the use of nimodipine could significantly reduce the rate of the poor outcome in patients with aSAH (RR =0.69, 95% confidence interval, 0.60–0.78, *p* < 0.01). There was no obvious heterogeneity in this study result (*p* = 0.16, I^2^ = 29%), fully demonstrating the stability of the conclusion. On the basis of this result, we further conducted multilevel subgroup analysis of the poor outcome, including mean age, sample size, number of study centers, and the route of administration. Interestingly, our important finding was that nimodipine use could significantly reduce the occurrence of the poor outcome in the subgroup of average age < 50 years (RR = 0.62, 95% confidence interval, 0.52–0.73, *p* < 0.01). However, nimodipine did not significantly ameliorate poor outcomes in subgroups of mean age of 50 years or more (RR = 0.85, 95% confidence interval, 0.71–1.03, *p* = 0.09 > 0.05). The statistical result difference between this mean age subgroup implied that nimodipine had dramatically different clinical effects on patients of different age ranges (*P* for interaction = 0.01 < 0.05), which, to some extent, was not consistent with long-term clinical observation and practice experience. To elucidate this contradictory result, our study further conducted a detailed quality assessment of included studies and then elaborately evaluated evidence recommendation levels of all outcome variables and their age subgroups by using the GRADE approach, respectively (15, 16). We finally found that the results of subgroup analysis with the age of < 50 years had the highest quality of evidence, and the recommended level was classified as strong recommendation. On the contrary, the evaluation results of patients with a mean age of 50 years or older showed a low quality of evidence, which implied that more in-depth analysis of large sample RCTs would most likely change these statistical results. All in all, our results did suggest that nimodipine could be used in patients with aSAH for reducing a poor good outcome, especially in patients younger than 50. Meanwhile, we did not recommend the clinical practice that nimodipine was not treated as a prophylactic drug for protecting against a poor outcome in patients with aSAH older than 50 years.

Two subgroups about the poor outcome were respectively analyzed according to the sample size and the number of research centers (Supplementary Table 5). The subgroup analyses results showed that the effect of nimodipine did not depend on the number of the samples and study centers, which further proved the stability of our conclusions that nimodipine did reduce the poor outcome in patients with aSAH. Furthermore, by the subgroup analysis of the drug administration route, we found that both oral and direct endovascular administrations could improve the patient outcomes (Supplementary Table 5), which was consistent with the previous reports (29). However, due to the limited number of studies included in this subgroup, this study did not identify the specific optimal drug dose and route of administration. Therefore, further investigating studies are still needed for guiding clinical practice in the future.

The results of this study on mortality are consistent with the research of Liu et al. (28). The difference from a previous study was that we further conducted a comprehensive subgroup analysis about the mortality, along with the evidence quality assessment and the recommendation grade evaluation for the mean age subgroup. No significantly statistical differences were found within the three subgroups of the route of administration, the sample size, and the number of research centers, except for the mean age subgroup analysis. If analyzed from a statistical-only perspective, nimodipine could significantly reduce patients' mortality. However, the evidence quality evaluation of both the overall mortality and the subgroups was evaluated as moderate and low, respectively. Therefore, more high-quality RCTS of nimodipine are needed to determine its efficacy on mortality in SAH in the future research.

Cerebral vasospasm often occurred about 4–14 days after aneurysm subarachnoid hemorrhage and was a leading cause of a poor outcome and death (30–32). Among the 13 RCTs included in our study, only six trials recorded in detail the incidence of cerebral vasospasm. We found significant statistical difference in the incidence of cerebral vasospasm between the nimodipine group and the control group. Moreover, the evidence grade of this result was evaluated as moderate, which meant more high-quality documents were needed to further confirm the preventive effect of nimodipine on cerebral vasospasm.

## Strengths and limitations

Our meta-analysis has several important strengths. Firstly, we conducted a comprehensive search of the mainstream databases and rigorously followed the recommendations of the Cochrane Collaboration and PRISMA statement. We looked up in detail the relevant websites and clinical trial registries for unpublished trials. Furthermore, our study performed four subgroup analyses of the primary outcome and yielded more stable and reliable statistical results. Finally, our team deliberately conducted an assessment of the quality of evidence by using the GRADE approach and developed accurate clinical recommendation grades for the primary outcome and the secondary outcome.

It must be acknowledged that there were also some limitations in our study. Firstly, the total number of original RCTs included in our meta-analysis was small, especially the proportion of updated articles in recent years. Secondly, due to the lack of long-term follow-up reports in most pieces of literature, the data in our study were limited and mainly derived from the short-term efficacy of nimodipine during patient hospitalization. In addition, the route of administration and the dose of nimodipine used in included trials varied considerably, which resulted in a predicament that this study could not determine how to use nimodipine most effectively. These uncertainties related with the treatment regiments of nimodipine in patients with aSAH needed more large RCTs for further investigation.

## Conclusion

This meta-analysis indicates that nimodipine can significantly reduce the incidence of poor outcome, mortality, and CVS in patients with aSAH. Meanwhile, we strongly recommend that patients with aSAH, especially those younger than 50 years old, should use nimodipine as early as possible to achieve a better clinical outcome.

## Data availability statement

The original contributions presented in the study are included in the article/Supplementary materials, further inquiries can be directed to the corresponding author/s.

## Author contributions

GH and GC determined the topic selection and wrote the main manuscript text. PP and YH organized the data and prepared all the tables. YA and ZS prepared all the figures. ZS and GL reviewed the manuscript. All authors listed have made a substantial, direct, and intellectual contribution to the work and approved it for publication.

## Funding

This project was sponsored by Research Project of Shenyang Bureau of Science and Technology (20-205-4-017), National Natural Science Foundation of China (81971133), Liaoning Key Research and Development Project (2019JH8/10300085 and 2021JH2/10300059), and LiaoNing Revitalization Talents Program (XLYC2002109).

## Conflict of interest

The authors declare that the research was conducted in the absence of any commercial or financial relationships that could be construed as a potential conflict of interest.

## Publisher's note

All claims expressed in this article are solely those of the authors and do not necessarily represent those of their affiliated organizations, or those of the publisher, the editors and the reviewers. Any product that may be evaluated in this article, or claim that may be made by its manufacturer, is not guaranteed or endorsed by the publisher.
