# Supplementary material for: Clinical effectiveness of nimodipine for the prevention of poor outcome after aneurysmal subarachnoid hemorrhage: A systematic review and meta-analysis
Source: Front Neurol. 2022 Sep 21;13:982498. doi: 10.3389/fneur.2022.982498 (PMC9533126; doi:10.3389/fneur.2022.982498)
Supplement: Supplementary file 1 [file Table_1.DOC]

Pubmed: **search strategy**

((randomized controlled trial[Publication Type] OR randomized[Title/Abstract] OR placebo[Title/Abstract]) AND (("Subarachnoid Hemorrhage"[Mesh]) OR ((((((((((((((((((((((((((((((SAH[Title/Abstract]) OR (SAHs[Title/Abstract])) OR (Hemorrhage, Subarachnoid[Title/Abstract])) OR (Hemorrhages, Subarachnoid[Title/Abstract])) OR (Subarachnoid Hemorrhages[Title/Abstract])) OR (Subarachnoid Hemorrhage, Aneurysmal[Title/Abstract])) OR (Aneurysmal Subarachnoid Hemorrhage[Title/Abstract])) OR (Aneurysmal Subarachnoid Hemorrhages[Title/Abstract])) OR (Hemorrhage, Aneurysmal Subarachnoid[Title/Abstract])) OR (Hemorrhages, Aneurysmal Subarachnoid[Title/Abstract])) OR (Subarachnoid Hemorrhages, Aneurysmal[Title/Abstract])) OR (Subarachnoid Hemorrhage, Spontaneous[Title/Abstract])) OR (Hemorrhage, Spontaneous Subarachnoid[Title/Abstract])) OR (Hemorrhages, Spontaneous Subarachnoid[Title/Abstract])) OR (Spontaneous Subarachnoid Hemorrhage[Title/Abstract])) OR (Spontaneous Subarachnoid Hemorrhages[Title/Abstract])) OR (Subarachnoid Hemorrhages, Spontaneous[Title/Abstract])) OR (Perinatal Subarachnoid Hemorrhage[Title/Abstract])) OR (Hemorrhage, Perinatal Subarachnoid[Title/Abstract])) OR (Hemorrhages, Perinatal Subarachnoid[Title/Abstract])) OR (Perinatal Subarachnoid Hemorrhages[Title/Abstract])) OR (Subarachnoid Hemorrhage, Perinatal[Title/Abstract])) OR (Subarachnoid Hemorrhages, Perinatal[Title/Abstract])) OR (Subarachnoid Hemorrhage, Intracranial[Title/Abstract])) OR (Hemorrhage, Intracranial Subarachnoid[Title/Abstract])) OR (Hemorrhages, Intracranial Subarachnoid[Title/Abstract])) OR (Intracranial Subarachnoid Hemorrhage[Title/Abstract])) OR (Intracranial Subarachnoid Hemorrhages[Title/Abstract])) OR (Subarachnoid Hemorrhages, Intracranial[Title/Abstract])))) AND (("Nimodipine"[Mesh]) OR ((((((((((((((((Bay e 9736[Title/Abstract]) OR (e 9736, Bay[Title/Abstract])) OR (Brainal[Title/Abstract])) OR (Calnit[Title/Abstract])) OR (Kenesil[Title/Abstract])) OR (Nymalize[Title/Abstract])) OR (Nimodipin Hexal[Title/Abstract])) OR (Hexal, Nimodipin[Title/Abstract])) OR (Nimodipin-ISIS[Title/Abstract])) OR (Nimodipin ISIS[Title/Abstract])) OR (Nimodipino Bayvit[Title/Abstract])) OR (Bayvit, Nimodipino[Title/Abstract])) OR (Nimotop[Title/Abstract])) OR (Remontal[Title/Abstract])) OR (Admon[Title/Abstract])) OR (Modus[Title/Abstract])))

**Embase: search strategy**

| #1   | subarachnoid AND ('hemorrhage'/exp OR hemorrhage) | 57,217 |  | | --- | --- | --- | | #2 | sah (subarachnoid hemorrhage)':ab,ti OR 'sahs (subarachnoid hemorrhage)':ab,ti OR 'hemorrhage, subarachnoid':ab,ti OR 'hemorrhages, subarachnoid':ab,ti OR 'subarachnoid hemorrhages':ab,ti OR 'subarachnoid hemorrhage, aneurysmal':ab,ti OR 'aneurysmal subarachnoid hemorrhage':ab,ti OR 'aneurysmal subarachnoid hemorrhages':ab,ti OR 'hemorrhage, aneurysmal subarachnoid':ab,ti OR 'hemorrhages, aneurysmal subarachnoid':ab,ti OR 'subarachnoid hemorrhages, aneurysmal':ab,ti OR 'subarachnoid hemorrhage, spontaneous':ab,ti OR 'hemorrhage, spontaneous subarachnoid':ab,ti OR 'hemorrhages, spontaneous subarachnoid':ab,ti OR 'spontaneous subarachnoid hemorrhage':ab,ti OR 'spontaneous subarachnoid hemorrhages':ab,ti OR 'subarachnoid hemorrhages, spontaneous':ab,ti OR 'perinatal subarachnoid hemorrhage':ab,ti OR 'hemorrhage, perinatal subarachnoid':ab,ti OR 'hemorrhages, perinatal subarachnoid':ab,ti OR 'perinatal subarachnoid hemorrhages':ab,ti OR 'subarachnoid hemorrhage, perinatal':ab,ti OR 'subarachnoid hemorrhages, perinatal':ab,ti OR 'subarachnoid hemorrhage, intracranial':ab,ti OR 'hemorrhage, intracranial subarachnoid':ab,ti OR 'hemorrhages, intracranial subarachnoid':ab,ti OR 'intracranial subarachnoid hemorrhage':ab,ti OR 'intracranial subarachnoid hemorrhages':ab,ti OR 'subarachnoid hemorrhages, intracranial':ab,ti | 8,408 | | #3 | #1 OR #2 | 57,295 | | #4 | nimodipine | 11,468 | | #5 | 'bay e 9736':ab,ti OR 'e 9736, bay':ab,ti OR 'brainal':ab,ti OR 'calnit':ab,ti OR 'kenesil':ab,ti OR 'nymalize':ab,ti OR 'nimodipin hexal':ab,ti OR 'hexal, nimodipin':ab,ti OR 'nimodipin-isis':ab,ti OR 'nimodipin isis':ab,ti OR 'nimodipino bayvit':ab,ti OR 'bayvit, nimodipino':ab,ti OR 'nimotop':ab,ti OR 'remontal':ab,ti OR 'admon':ab,ti OR 'modus':ab,ti | 1,832 | | #6 | #4 OR #5 | 13,187 | | #7 | 'randomized controlled trial':ab,ti OR 'randomized':ab,ti OR 'placebo':ab,ti OR 'rct':ab,ti | 1,067,672 | | #8 | #3 AND #6 AND #7 | 257 | |  |  |
| --- | --- | --- | --- | --- | --- | --- | --- | --- | --- | --- | --- | --- | --- | --- | --- | --- | --- | --- | --- | --- | --- | --- | --- | --- | --- | --- |
| **Cochrane library** **search strategy**   | 1 | Subarachnoid Hemorrhage | 2393 | | --- | --- | --- | | 2 | (SAH (Subarachnoid Hemorrhage)):ab,ti,kw OR(SAHs (Subarachnoid Hemorrhage)):ab,ti,kw OR(Hemorrhage, Subarachnoid):ab,ti,kw OR(Hemorrhages, Subarachnoid):ab,ti,kw OR(Subarachnoid Hemorrhages):ab,ti,kw OR(Subarachnoid Hemorrhage, Aneurysmal):ab,ti,kw OR(Aneurysmal Subarachnoid Hemorrhage):ab,ti,kw OR(Aneurysmal Subarachnoid Hemorrhages):ab,ti,kw OR(Hemorrhage, Aneurysmal Subarachnoid):ab,ti,kw OR(Hemorrhages, Aneurysmal Subarachnoid):ab,ti,kw OR(Subarachnoid Hemorrhages, Aneurysmal):ab,ti,kw OR(Subarachnoid Hemorrhage, Spontaneous):ab,ti,kw OR(Hemorrhage, Spontaneous Subarachnoid):ab,ti,kw OR(Hemorrhages, Spontaneous Subarachnoid):ab,ti,kw OR(Spontaneous Subarachnoid Hemorrhage):ab,ti,kw OR(Spontaneous Subarachnoid Hemorrhages):ab,ti,kw OR(Subarachnoid Hemorrhages, Spontaneous):ab,ti,kw OR(Perinatal Subarachnoid Hemorrhage):ab,ti,kw OR(Hemorrhage, Perinatal Subarachnoid):ab,ti,kw OR(Hemorrhages, Perinatal Subarachnoid):ab,ti,kw OR(Perinatal Subarachnoid Hemorrhages):ab,ti,kw OR(Subarachnoid Hemorrhage, Perinatal):ab,ti,kw OR(Subarachnoid Hemorrhages, Perinatal):ab,ti,kw OR(Subarachnoid Hemorrhage, Intracranial):ab,ti,kw OR(Hemorrhage, Intracranial Subarachnoid):ab,ti,kw OR(Hemorrhages, Intracranial Subarachnoid):ab,ti,kw OR(Intracranial Subarachnoid Hemorrhage):ab,ti,kw OR(Intracranial Subarachnoid Hemorrhages):ab,ti,kw OR(Subarachnoid Hemorrhages, Intracranial):ab,ti,kw | 2170 | | 3 | #1 OR #2 | 2400 | | 4 | (nimodipine):ab,ti,kw OR(Bay e 9736):ab,ti,kw OR(e 9736, Bay):ab,ti,kw OR(Brainal):ab,ti,kw OR(Calnit):ab,ti,kw OR(Kenesil):ab,ti,kw OR(Nymalize):ab,ti,kw OR(Nimodipin Hexal):ab,ti,kw OR(Hexal, Nimodipin):ab,ti,kw OR(Nimodipin-ISIS):ab,ti,kw OR(Nimodipin ISIS):ab,ti,kw OR(Nimodipino Bayvit):ab,ti,kw OR(Bayvit, Nimodipino):ab,ti,kw OR(Nimotop):ab,ti,kw OR(Remontal):ab,ti,kw OR(Admon):ab,ti,kw OR(Modus):ab,ti,kw | 923 | | 5 | (randomized controlled trial):ab,ti,kw OR(randomized):ab,ti,kw OR(placebo):ab,ti,kw OR(RCT):ab,ti,kw | 1112696 | | 6 | #3 AND #4 AND #5 | 139 | |  |  |
|  |  |  |
|  |  |  |
| Scopus: **search strategy**  ( TITLE-ABS-KEY ( "Subarachnoid Hemorrhage"  OR  "SAH "  OR  "Subarachnoid Hemorrhage"  OR  "SAHs "  OR  "Subarachnoid Hemorrhage"  OR  "Hemorrhage, Subarachnoid"  OR  "Hemorrhages, Subarachnoid"  OR  "Subarachnoid Hemorrhages"  OR  "Subarachnoid Hemorrhage, Aneurysmal"  OR  "Aneurysmal Subarachnoid Hemorrhage"  OR  "Aneurysmal Subarachnoid Hemorrhages"  OR  "Hemorrhage, Aneurysmal Subarachnoid"  OR  "Hemorrhages, Aneurysmal Subarachnoid"  OR  "Subarachnoid Hemorrhages, Aneurysmal"  OR  "Subarachnoid Hemorrhage, Spontaneous"  OR  "Hemorrhage, Spontaneous Subarachnoid"  OR  "Hemorrhages, Spontaneous Subarachnoid"  OR  "Spontaneous Subarachnoid Hemorrhage"  OR  "Spontaneous Subarachnoid Hemorrhages"  OR  "Subarachnoid Hemorrhages, Spontaneous"  OR  "Perinatal Subarachnoid Hemorrhage"  OR  "Hemorrhage, Perinatal Subarachnoid"  OR  "Hemorrhages, Perinatal Subarachnoid"  OR  "Perinatal Subarachnoid Hemorrhages"  OR  "Subarachnoid Hemorrhage, Perinatal"  OR  "Subarachnoid Hemorrhages, Perinatal"  OR  "Subarachnoid Hemorrhage, Intracranial"  OR  "Hemorrhage, Intracranial Subarachnoid"  OR  "Hemorrhages, Intracranial Subarachnoid"  OR  "Intracranial Subarachnoid Hemorrhage"  OR  "Intracranial Subarachnoid Hemorrhages"  OR  "Subarachnoid Hemorrhages, Intracranial" )  AND  TITLE-ABS-KEY ( "nimodipine"  OR  "Bay e 9736"  OR  "e 9736, Bay"  OR  "Brainal"  OR  "Calnit"  OR  "Kenesil"  OR  "Nymalize"  OR  "Nimodipin Hexal"  OR  "Hexal, Nimodipin"  OR  "Nimodipin-ISIS"  OR  "Nimodipin ISIS"  OR  "Nimodipino Bayvit"  OR  "Bayvit, Nimodipino"  OR  "Nimotop"  OR  "Remontal"  OR  "Admon" )  AND  TITLE-ABS-KEY ( "randomized controlled trial"  OR  "randomized"  OR  "placebo"  OR  "RCT" ) ) |  |  |
|  |  |  |
|  |  |  |
|  |  |  |
|  |  |  |
|  |  |  |
|  |  |  |
|  |  |  |
|  |  |  |
|  |  |  |
|  |  |  |
|  |  |  |
