# Supplementary material for: Clinical effectiveness of nimodipine for the prevention of poor outcome after aneurysmal subarachnoid hemorrhage: A systematic review and meta-analysis
Source: Front Neurol. 2022 Sep 21;13:982498. doi: 10.3389/fneur.2022.982498 (PMC9533126; doi:10.3389/fneur.2022.982498)
Supplement: Supplementary file 2 [file Table_2.DOC]

| Outcomes | Definitions |
| --- | --- |
| Poor outcome | Severe disability combined with deaths from any causes |
| Mortality | Just count the number of deaths |
| Vasospasm | Arterial diameter was reduced by more than 50% on angiography |
