# Supplementary material for: Clinical effectiveness of nimodipine for the prevention of poor outcome after aneurysmal subarachnoid hemorrhage: A systematic review and meta-analysis
Source: Front Neurol. 2022 Sep 21;13:982498. doi: 10.3389/fneur.2022.982498 (PMC9533126; doi:10.3389/fneur.2022.982498)
Supplement: Supplementary file 3 [file Table_3.DOC]

| Trial | Country | Mean age (years) | Female rate(%) | Adiministration | Intervention | Control | Follow-up |
| --- | --- | --- | --- | --- | --- | --- | --- |
| J.Philippon 1986 | France | 45.0 | 57.14 | oral | nimodipine (60mmg every four hours) | placebo | 1-month |
| G.Neil-Dwyer 1987 | Britain | 48.3 | 66.00 | oral | nimodipine(60mmg every four hours) | placebo | 3-month |
| Michel Jan 1988 | France | 47.9 | 53.54 | intravenous | nimodipine(2mg/h) | placebo | 6-month |
| Edward Mee 1988 | Britain | 50.0 | 54.00 | oral | nimodipine(30mg/day) | placebo | 3-month |
| Juha 1988 | Finland | 44.4 | 51.97 | intravenous | nimodipine(0.5μg/kg/min) | placebo | 3-month |
| K.C.Petruk 1988 | Canada | 55.0 | 66.88 | oral | nimodipine(90mmg every four hours) | placebo | 3-month |
| Pickard, J. D. 1989 | Britain | 47.0 | 60.11 | oral | nimodipine (60mmg every four hours) | placebo | 3-month |
| Desbordes, J. M. 1989 | France | 47.9 | 51.97 | intravenous | nimodipine(2mg/h) | placebo | 3-month |
| Ohman, J. 1991 | Finland | 45.2 | 51.37 | intravenous | nimodipine(0.5μg/kg/min) | placebo | 1.4-year |
| Han, D. H. 2006 | China | 50.1 | 61.11 | intravenous | nimodipine(1 mg/hr) | fasudil (30mg, three times a day) | 1-month |
| van den Bergh 2006 | Germany | 53.0 | 58.65 | intravenous | nimodipine(48mg/kg daily) | magnesium(40mg/kg daily) | 1-year |
| Selbach, M. 2019 | Germany | 49.0 | 68.29 | intra-arterial | nimodipine(between 0.5 and 1.2mg/h) | placebo | 6-month |
| Yindeedej, V. 2021 | Thailand | 56.1 | 73.53 | intra-arterial | nimodipine(1-5mg in each vessel, at the rate of 1 mg per 5minutes) | placebo | 6-month |
