# Supplementary material for: Clinical effectiveness of nimodipine for the prevention of poor outcome after aneurysmal subarachnoid hemorrhage: A systematic review and meta-analysis
Source: Front Neurol. 2022 Sep 21;13:982498. doi: 10.3389/fneur.2022.982498 (PMC9533126; doi:10.3389/fneur.2022.982498)
Supplement: Supplementary file 4 [file Table_4.DOCX]

**Author(s):**
**Date:** 2022-06-20
**Question:** nimodipine vs control for Subarachnoid Hemorrhage
**Settings:**
**Bibliography:** . Nimodipine for Subarachnoid Hemorrhage. Cochrane Database of Systematic Reviews [Year], Issue [Issue].

| **Quality assessment** | | | | | | | **No of patients** | | **Effect** | | **Quality** | **Importance** |
| --- | --- | --- | --- | --- | --- | --- | --- | --- | --- | --- | --- | --- |
|  |  |  |  |  |  |  |  |  |  |  |  |  |
| **No of studies** | **Design** | **Risk of bias** | **Inconsistency** | **Indirectness** | **Imprecision** | **Other considerations** | **Nimodipine** | **Control** | **Relative (95% CI)** | **Absolute** |  |  |
| **disability rate** | | | | | | | | | | | | |
| 13 | randomised trials | no serious risk of bias | no serious inconsistency | no serious indirectness | no serious imprecision | none | 232/873  (26.6%) | 329/854  (38.5%) | RR 0.69 (0.6 to 0.78) | 119 fewer per 1000 (from 85 fewer to 154 fewer) | ⊕⊕⊕⊕ HIGH | CRITICAL |
| **disability subgroup mean of age - mean of age >=50** | | | | | | | | | | | | |
| 5 | randomised trials | no serious risk of bias | no serious inconsistency | no serious indirectness | very serious^1^ | none | 81/219  (37%) | 103/229  (45%) | RR 0.85 (0.71 to 1.03) | 67 fewer per 1000 (from 130 fewer to 13 more) | ⊕⊕OO LOW | CRITICAL |
| **disability subgroup mean of age - mean of age <50** | | | | | | | | | | | | |
| 8 | randomised trials | no serious risk of bias | no serious inconsistency | no serious indirectness | no serious imprecision | none | 151/654  (23.1%) | 226/625  (36.2%) | RR 0.62 (0.52 to 0.73) | 137 fewer per 1000 (from 98 fewer to 174 fewer) | ⊕⊕⊕⊕ HIGH | CRITICAL |
| **mortality rate** | | | | | | | | | | | | |
| 13 | randomised trials | no serious risk of bias | serious^2^ | no serious indirectness | no serious imprecision | none | 112/873  (12.8%) | 181/854  (21.2%) | RR 0.5 (0.32 to 0.78) | 106 fewer per 1000 (from 47 fewer to 144 fewer) | ⊕⊕⊕O MODERATE | CRITICAL |
| **Mortality subgroup mean of age - mean of age >=50** | | | | | | | | | | | | |
| 5 | randomised trials | no serious risk of bias | no serious inconsistency | no serious indirectness | very serious^1^ | none | 35/219  (16%) | 36/229  (15.7%) | RR 1.08 (0.73 to 1.59) | 13 more per 1000 (from 42 fewer to 93 more) | ⊕⊕OO LOW | CRITICAL |
| **Mortality subgroup mean of age - mean of age<50** | | | | | | | | | | | | |
| 8 | randomised trials | no serious risk of bias | serious^2^ | no serious indirectness | no serious imprecision | reporting bias^3^ | 77/654  (11.8%) | 145/625  (23.2%) | RR 0.5 (0.39 to 0.64) | 116 fewer per 1000 (from 84 fewer to 142 fewer) | ⊕⊕OO LOW | CRITICAL |
| **cerebral vasospasm** | | | | | | | | | | | | |
| 6 | randomised trials | no serious risk of bias | serious^2^ | no serious indirectness | no serious imprecision | none | 86/308  (27.9%) | 129/285  (45.3%) | RR 0.68 (0.46 to 0.99) | 145 fewer per 1000 (from 5 fewer to 244 fewer) | ⊕⊕⊕O MODERATE | IMPORTANT |

^1^ RR的95%置信区间包括了等效线，且样本量较小
^2^ 存在中度一致性，I^2^>50%
^3^ 存在发表偏倚
