# Supplementary material for: Clinical effectiveness of nimodipine for the prevention of poor outcome after aneurysmal subarachnoid hemorrhage: A systematic review and meta-analysis
Source: Front Neurol. 2022 Sep 21;13:982498. doi: 10.3389/fneur.2022.982498 (PMC9533126; doi:10.3389/fneur.2022.982498)
Supplement: Supplementary file 5 [file Table_5.DOC]

|  | | | | | | |
| --- | --- | --- | --- | --- | --- | --- |
| **Subgroup title** | **No. of trials** | **No. of participants** | **I^2^(%)** | **Risk ratio(95%Cl)** | **P** | **P for interaction** |
| Overall | 13 | 1727 | 29 | 0.71(0.61-0.83) | ＜0.001 | — |
| No. of centers |  |  |  |  |  |  |
| Single-center | 9 | 765 | 0 | 0.67(0.53-0.85) | 0.001 | 0.77 |
| Multi-center | 4 | 962 | 70 | 0.70(0.53-0.93) | 0.01 |
| Sample size |  |  |  |  |  |  |
| ≥80 | 7 | 1376 | 45 | 0.70(0.61-0.81) | ＜0.001 | 0.54 |
| ＜80 | 6 | 351 | 13 | 0.63(0.47-0.85) | 0.002 |
| Administratons |  |  |  |  |  |  |
| Oral | 5 | 878 | 73 | 0.61(0.41-0.91) | 0.02 | 0.64 |
| Vessel | 8 | 849 | 0 | 0.71(0.58-0.87) | ＜0.001 |
| Mean of age |  |  |  |  |  |  |
| ≥50 | 5 | 448 | 0 | 0.85(0.71-1.03) | 0.09 | 0.01* |
| ＜50 | 8 | 1279 | 0 | 0.62(0.52-0.73) | ＜0.001 |

*Statistically significant.
