# Supplementary material for: Clinical effectiveness of nimodipine for the prevention of poor outcome after aneurysmal subarachnoid hemorrhage: A systematic review and meta-analysis
Source: Front Neurol. 2022 Sep 21;13:982498. doi: 10.3389/fneur.2022.982498 (PMC9533126; doi:10.3389/fneur.2022.982498)
Supplement: Supplementary file 6 [file Table_6.DOC]

| Table. Subgroup analysis of the effect of nimodipine on mortality | | | | | | |
| --- | --- | --- | --- | --- | --- | --- |
| **Subgroup title** | **No. of trials** | **No. of participants** | **I^2^(%)** | **Risk ratio(95%Cl)** | **P** | **P for interaction** |
| Overall | 13 | 1727 | 62 | 0.50(0.32-0.78) | 0.002 | — |
| No. of centers |  |  |  |  |  |  |
| Single-center | 9 | 765 | 0 | 0.47(0.30-0.72) | <0.001 | 0.93 |
| Multi-center | 4 | 962 | 86 | 0.54(0.26-1.12) | <0.001 |
| Sample size |  |  |  |  |  |  |
| ≥80 | 7 | 1376 | 76 | 0.55(0.32-0.95) | 0.03 | 0.38 |
| <80 | 6 | 351 | 0 | 0.35(0.17-0.70) | 0.003 |
| Administratons |  |  |  |  |  |  |
| Oral | 5 | 878 | 65 | 0.69(0.37-1.27) | 0.23 | 0.16 |
| Vessel | 8 | 849 | 26 | 0.39(0.27-0.56) | <0.001 |
| Mean of age |  |  |  |  |  |  |
| ≥50 | 5 | 448 | 25 | 1.08(0.73-1.59) | 0.71 | 0.001^*^ |
| <50 | 8 | 1279 | 51 | 0.42(0.26-0.66) | <0.001 |

^*^Statistically significant.
